# Risk Factors for Prenatal Anxiety in European Women: A Review

**DOI:** 10.3390/jcm14093248

**Published:** 2025-05-07

**Authors:** Alba Val, Cristina M. Posse, M. Carmen Míguez

**Affiliations:** Faculty of Psychology, Department of Clinical Psychology and Psychobiology, Institute of Psychology (IPsiUS), University of Santiago de Compostela, Campus Vida, 15782 Santiago de Compostela, Spain; alba.val@rai.usc.es (A.V.); cristinamaria.posse.cepeda@usc.es (C.M.P.)

**Keywords:** anxiety, pregnancy, prenatal, risk factors, women, review

## Abstract

**Background:** Prenatal anxiety is a common problem affecting a large number of women. The presence of anxiety during pregnancy is associated with adverse consequences for both the mother and the baby. The main objective of this review was to determine the risk factors associated with anxiety during pregnancy in European women. Specifically, we wanted to know if these factors are the same as those found in other continents and if they are similar to those associated with depression during this stage. **Methods:** A literature review was carried out on studies that were published in the last 10 years in the PsycInfo, Medline, and SCOPUS databases. Thirteen studies were selected for the purposes of this review. **Results:** Sociodemographic risk factors associated with a higher level of anxiety during pregnancy included having a lower educational level and socioeconomic status. Obstetric and pregnancy-related risk factors included having had complications during pregnancy. Having a history of mental health problems, low social support, high levels of stress, and being exposed to adverse life events were the most relevant psychological factors for presenting prenatal anxiety. Furthermore, these factors are largely common to those associated with prenatal anxiety in other continents of the world and to those associated with prenatal depression. **Conclusions**: This review shows that there are multiple factors that contribute to women experiencing prenatal anxiety. Most can be identified at the beginning of pregnancy, and some factors, such as psychological ones, are potentially modifiable. This underlines the importance of carrying out a proper screening for anxiety during pregnancy in order to prevent its onset or treat it appropriately. Furthermore, the fact that risk factors are common for both prenatal anxiety and depression implies that the same intervention could reduce the probability of the onset of both pathologies and the possible consequences associated with them.

## 1. Introduction

Traditionally, pregnancy was considered a time of happiness for women [1], and it was believed to be a protective factor against the onset of mental disorders. However, it has been shown to be a period of vulnerability for developing a mental disorder [2,3]. Specifically, it is estimated that 20% of women may develop a mental disorder during the perinatal period, primarily anxiety and depression [4,5]. Compared to pregnancy, the postnatal period has received more attention in research. One reason for this is the wrong idea that women are “hormonally protected” from mental disorders during pregnancy [6]. Likewise, women may feel hesitant to share symptoms of sadness or anxiety due to the stigma associated with suffering from anxiety and/or depression during pregnancy, as it has been socially assumed that this should be a happy time [7]. Additionally, at the professional level, there is a tendency to focus more on physical health (both maternal and fetal) during pregnancy rather than mental health [8]. Furthermore, many pregnant women experience characteristic symptoms and/or complaints of anxiety or depression [9], such as fatigue, loss of energy, or sleep disturbances. Therefore, it can be challenging to distinguish between common pregnancy symptoms and those specific to anxiety or depression [10,11].

In the last few years, the interest in the prenatal phase has increased, which has shown that anxiety during pregnancy is common [12]. Its prevalence is estimated to be between 15% and 23% [13,14,15,16]. Some studies suggest that the prevalence of anxiety is higher during pregnancy than in the postpartum period [10,17], and that during pregnancy, it could even be more prevalent than depression [13,18]. However, research aimed at detecting and treating anxiety in this phase has been limited [19], and it has to be taken into consideration that prenatal anxiety has been related to adverse effects for the mother and her children. Regarding the mothers, it has been associated with the increased probability of developing depression in postpartum [20], a major risk of preeclampsia, obstetric complications, and attachment difficulties [21,22,23]. As for newborns, it is associated with lower gestational age, lower birth weight, and poor cognitive development, among others [24,25]. These findings suggest the importance of early detection and intervention of prenatal anxiety to promote the well-being of mothers and children.

Although there is increasing research on mental health in mothers, most research has centered on studying depression at the prevalence level [26,27], consequences [28,29], and/or on associated risk factors [29,30]. Detecting the factors associated with the presence of a mental health disorder is important for the prevention, detection, and effective treatment of such a disorder. Therefore, understanding the risk factors associated with prenatal anxiety will allow us to identify those women who are more likely to develop anxiety during pregnancy, with the aim of implementing preventive strategies to reduce the likelihood of such symptoms appearing.

Considering the high comorbidity between depression and anxiety during pregnancy [12], it would be interesting to determine whether both disorders share risk factors, as this would allow for simultaneous detection, prevention, and treatment. In fact, some studies suggest that a transdiagnostic intervention could be more beneficial than interventions specific to each disorder [31].

Until now, the studies that researched risk factors related to anxiety during pregnancy have been scarce. Moreover, previous reviews have analyzed risk factors of prenatal anxiety combined with depression [32], have focused exclusively on the risk factors associated with the different anxiety disorders [33], or gathered information on risk factors regardless of the country from which the study originates [34]. There are no studies that aimed to investigate the risk factors associated with prenatal anxiety in women of different continents, and it must be taken into consideration that cultural differences may condition the presence of such a pathology.

Even though it is known that in Europe anxiety affects a large number of women during pregnancy [16], no specific review has been conducted on risk factors associated with prenatal anxiety in women of this continent. Understanding the risk factors can contribute to the development of appropriate screening strategies and adequate preventive interventions to detect and treat anxiety in European pregnant women. Furthermore, it could prevent the presence of anxiety from extending into the postpartum period, with the consequences that this would entail for both mothers and their children [35]. Therefore, the main objective of this review is to identify the main risk factors associated with the presence of prenatal anxiety in Europe, through a review of the literature published in the last 10 years. Focusing on a specific continent permits us to know if risk factors associated with prenatal anxiety in European women are the same found in other continents, or if there are factors specifically associated with the European continent. Another important aspect is to know if the factors related to prenatal anxiety are similar to the ones associated with depression during this stage.

## 2. Materials and Methods

**Figure 1:**
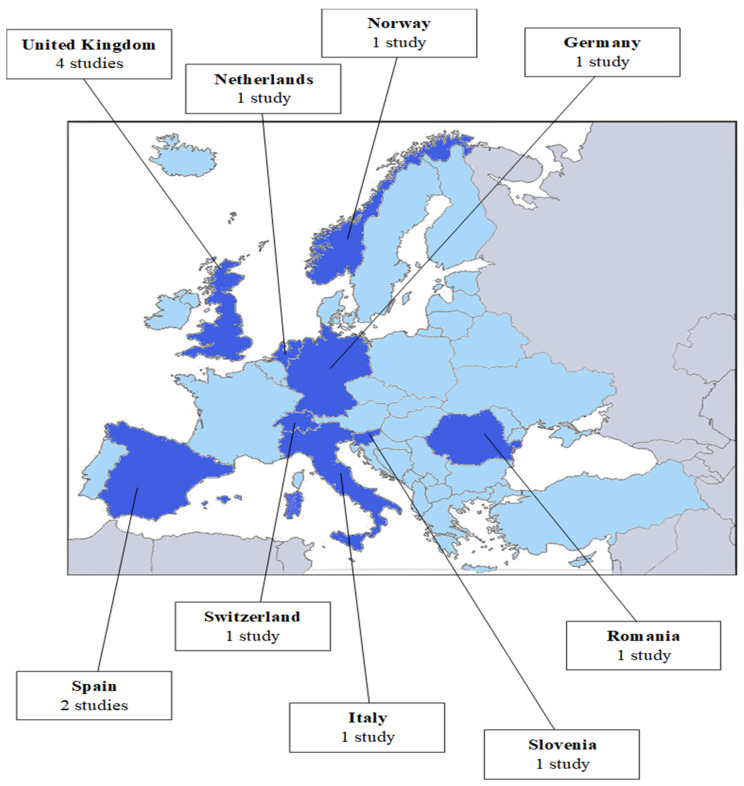
Countries included in the review. Note. Dark blue: European countries for which studies of risk factors for prenatal anxiety were found; Light blue: European countries for which no studies of risk factors for prenatal anxiety were found; Gray: non-European countries.

This study’s selection process was carried out following the recommendations of the Preferred Reporting Items for Systematic reviews and Meta-Analyses: PRISMA statement [36]. Thus, the articles had to meet the following inclusion criteria: (1) articles published between 1 January 2015 and 1 January 2025 (this temporal criterion has been established with the objective of providing current data from the scientific literature; (2) written in English; (3) containing the search keywords in the title and/or abstract; (4) assessing anxious symptomatology and/or generalized anxiety disorder (GAD); and (5) with a sample of women from the European continent. Therefore, the sample of women included refers to persons residing in that country, regardless of their place of origin and ethnicity. Specifically, 4 studies [15,37,38,39] included immigrant women, and 3 [40,41,42] included women of different ethnicities. However, they were required to write and speak fluently the language of the European country in which they resided. Moreover, in all cases, they represented a small number within the total sample. All the countries that are part of the European Union have been considered as the European continent (Figure 1). The United Kingdom has also been included, as it has been part of the European Union until 2021. The countries that are part of the European Free Trade Association (EFTA), such as Switzerland, Iceland, Liechtenstein, and Norway, have also been included. Studies that were not published in English and were not scientific studies (e.g., theses and book chapters) were excluded. Additionally, studies evaluating pregnancy-specific anxiety were excluded, since it is known that it is a different entity from the diagnostic and continuous measures of general anxiety, as it is a specific type of anxiety characterized by fears and/or concerns about pregnancy [37]; those that assessed anxiety in conjunction with other emotional symptomatology (e.g., comorbidity between anxiety and depression), those focused on specific anxiety disorders (e.g., panic disorder and post-traumatic stress disorder) or other mental disorders (e.g., eating disorders), studies including non-representative samples (e.g., twin pregnancies, war veterans, or families without resources), and other reviews.

### 2.1. Search Strategy

A literature review was conducted in the PsycINFO, Medline, and SCOPUS databases on existing publications from the last 10 years regarding risk factors associated with anxiety during the prenatal stage. The reference lists of the retrieved articles were also examined. The keywords used for the search were combined as follows: “risk factors” (abstract/title) OR “variables associated” (abstract/title) AND “antenatal anxiety” (abstract/title) OR “anxiety during pregnancy” (abstract/title). Each database was individually searched by title and abstract (Supplementary Table S1).

### 2.2. Strategy for the Selection of Studies and Analysis of Results

**Figure 2:**
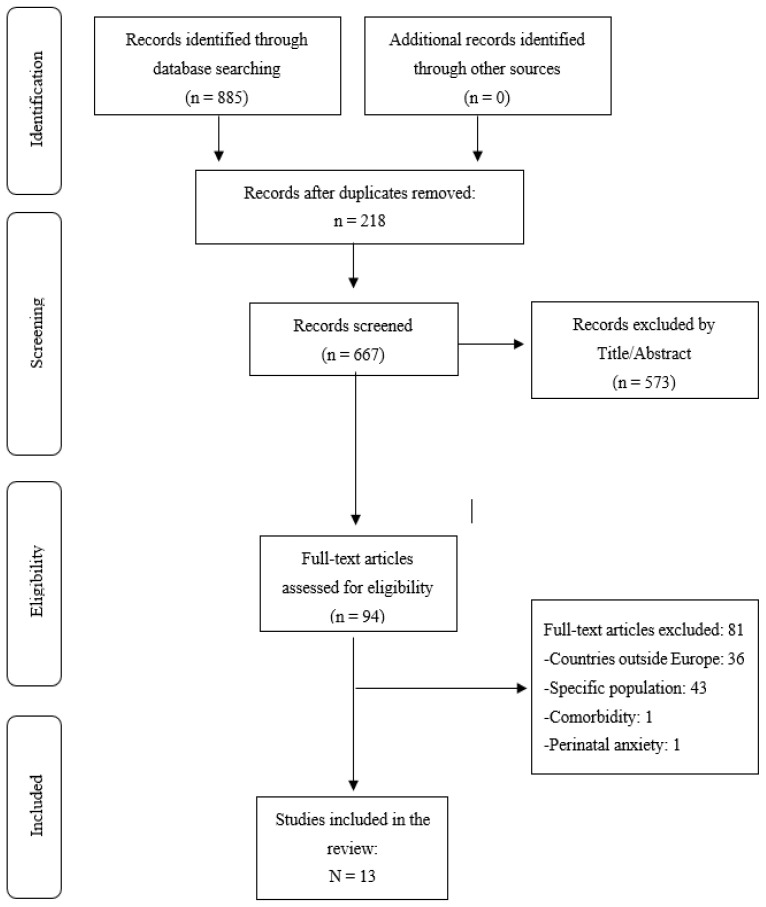
PRISMA flow diagram for the scoping review process.

To select the articles, the first author filtered each article by title and abstract, thus retrieving articles that met the inclusion criteria. The other authors independently screened one-third of the articles in the electronic search. Any disagreements regarding study inclusion were resolved through discussion among the authors. All publications that met the search criteria were transferred to Refworks, and all repeated publications were removed. The researchers read the titles and abstracts of the initially selected articles and then read the full texts of the articles that passed this stage of the process. The PRISMA flow diagram for the scoping review in Figure 2 summarizes the study selection process.

The first author extracted the following data from each article: author names; year of publication; risk factors associated with prenatal anxiety; country/region in which the study was conducted; and exclusion criteria. 

## 3. Results

### 3.1. Study Selection

A total of 885 potential scientific publications were identified in the initial search strategy: PsycINFO (*n* = 474), Medline (*n* = 285), and SCOPUS (*n* = 126). The manual search did not identify any additional records. After the removal of duplicate publications (*n* = 218), 667 publications were reviewed. A systematic search was then carried out by screening the retrieved studies. A series of inclusion and exclusion criteria were used to guide the selection process. On the basis of title and abstract, 573 articles were excluded, including 8 systematic reviews whose reference lists were searched. Of the 94 publications reviewed by full text, 81 publications were excluded (belonged to countries outside Europe, *n* = 36; belonged to a specific population, *n* = 43; entire perinatal period, *n* = 1; analyzed comorbidity, *n* = 1). Therefore, 13 articles remained to be included in the review.

All the selected studies met the quality criteria evaluated using the Mixed Methods Assessment Tool (MMAT) [43], through two general screening questions and five specific questions depending on the design of each study. All pre-screened studied were considered eligible and of good quality. The authors then read each article in full text, extracting the information of interest for the review. Once the results were selected, they were compiled in a table format.

### 3.2. Characteristics of Selected Studies

The findings are presented in Table 1 and Table 2. Of the 13 studies that met the inclusion criteria, six were cross-sectional, four were cohort studies, and three were longitudinal studies. Of the six cross-sectional studies, three assessed women regardless of their gestational age, two focused on the second and third trimesters, and one on the first trimester. Regarding the cohort studies, three assessed women in the first trimester of pregnancy, one in the second, and one in the third. Only one study was that analyzed the risk factors associated with prenatal anxiety by trimester [15]. The limited availability of studies that separately identify the risk factors associated with prenatal anxiety by trimester may provide less precise information about these variables, as each trimester of pregnancy has specific characteristics, since physical changes, medical tests, and concerns faced by pregnant women vary throughout gestation. Therefore, variables that may be significantly related to prenatal anxiety in a given trimester (e.g., being in the first trimester and having had previous miscarriages) may not be if women are assessed in a different trimester.

The United Kingdom is the country that provides the most research on risk factors associated with perinatal anxiety (*n* = 4), followed by Italy and Spain (*n* = 2 each). Slovenia, the Netherlands, Norway, Romania, and Germany have each provided one study.

The samples of the studies were obtained, in most cases, from women who attended their pregnancy follow-up check-ups in their health centers and/or hospitals of reference; however, some conducted these consultations via videoconference due to the COVID-19 pandemic. A great variability in the sample size used was found, ranging between 84 [38] and 7824 women [41]. The total number of women in the 13 studies included was 170.006.

Variability was observed in defining and assessing anxiety because different assessment instruments were used. More specifically, 10 studies assessed prenatal anxiety using self-report questionnaires, with the State-Trait Anxiety Inventory being the most commonly used (*n* = 5), although in different countries [37,38,44], different cut-off points (≥40; >45; ≥45) were used. In all cases, the cut-off point recommended in other studies was used to assess prenatal anxiety. Only one study [45] used a clinical interview (CIDI-V) for the diagnosis of Generalized Anxiety Disorder. This interview is based on the criteria of the International Classification of Diseases (ICD-10). It must be taken into consideration that women can over- or under-estimate their responses in a self-reported questionnaire depending on their believes, perceptions, culture, and stigmatization of mental health in their communities. Specifically, it was found that in cultures with a traditional or more religious family structure, pregnant women tended to report fewer symptoms of depression [46]. This tendency to hide emotional distress could be extrapolated to European countries with more traditional cultures, such as Turkey or Romania. In fact, in a study carried out in Romania [47], they point out that in Eastern European countries there is no antenatal screening for mental health conditions, nor is there any screening in the postpartum period, highlighting the lack of attention to mental health in those countries with more traditional cultures and fewer resources.

The literature analysis showed that the risk factors for prenatal anxiety can be sociodemographic, obstetric, and/or pregnancy-related, as well as psychological. Some lifestyle-related variables, such as weight or substance use, were also mentioned in some studies. Additionally, it has been observed that sociodemographic variables have been the most investigated. Specifically, 50 results have been reported on these variables, compared to 17 results on obstetric and/or pregnancy-related factors and 18 on psychological risk factors. The significance level of the association (*p*-value) and the odds ratio of the predictor variables are provided when studies include this information, aiming to show the strength of the association between variables.

**Table 1:** Characteristics of the included studies (N = 13).

Study	Anxiety Measures	Sample Size (*n*)	Study Design	Regression Analysis
Martini et al. (2015) Germany [45]	CIDI-V	306	Longitudinal Prospective	Not specified
Podvornik et al. (2015) Slovenia [49]	STAI ≥ 45	348	Cross-sectional (1st, 2nd and 3rd T)	Bivariate
van de Loo et al. (2018) Netherlands [39]	HADS-A ≥ 8	2897	Longitudinal	Multivariate
Soto-Balbuena et al. (2018) Spain [15]	GAD-7 ≥ 7	932	Longitudinal	Not specified
Sharapova et al. (2018) Switzerland [38]	STAI-S ≥ 45	84	Cohort (3rd T)	Multivariate
Osnes et al. (2019) Norway [50]	SCL-A ≥ 18	1563	Cross-sectional (2nd y 3rd T)	Multivariate
González-Mesa et al. (2019) Spain [37]	STAI > 45	514	Cohort (1st T)	Bivariate and Multivariate
Cena et al. (2020) Italy [44]	STAI-S ≥ 40	1143	Cross-sectional (2nd y 3rd T)	Bivariate and Multivariate
Insan et al. (2020) United Kingdom [41]	GHQ > 6	7824	Cohort (2nd T)	Multivariate
Savory et al. (2021) United Kingdom [42]	GAD-7 ≥ 10	302	Cohorts (1st T)	Multivariate
Koyucu et al. (2021) United Kingdom [48]	DASS	729	Cross-sectional Pregnancy	Multivariate
Filippetti et al. (2022) United Kingdom [40]	STAI-S ≥ 40	150	Cross-sectional Pregnancy	Multivariate
Răchită et al. (2023) Romania [47]	HADS-A > 11 HAMA > 20	215	Cross-sectional (3rd T)	Multivariate

CIDI-V: Anxiety Composite International Diagnostic Interview; STAI: State Trait Anxiety Inventory; STAI-S: State Anxiety Subscale of the State Trait Anxiety Inventory; GAD-7: Generalized Anxiety Disorder 7-item Scale; SCL-A: anxiety dimension from the Hopkins Symptom Check List-25; GHQ: General Health Questionnaire; DASS: Depression Anxiety Stress Scale; HADS-A: Anxiety Subscale of the Hospital Anxiety Depression Scale; HAMA: Hamilton Anxiety Scale; T: Trimester; n: number of participants.

**Table 2:** Risk factors for prenatal anxiety in European women.

Authors and Country	Sociodemographic Variables	Obstetric Variables	Psychological Variables
Martini et al. (2015) Germany [45]	Unsatisfactory relationship *^1^ 0.98		-History of anxiety and/or depression *^1^ 14.31/1.79 -Experiencing trauma and/or sexual trauma *^1^ 3.15/2.96 -Low self-esteem *^1^ 0.89 -Low social support *^1^ 0.44
Podvornik et al. (2015) Slovenia [49]	Lower income level (state and trait) ^r^ 0.12/0.22 Lower educational level (trait) ^r^ 0.19		
van de Loo et al. (2018) Netherlands [39]	Age < 30 -yr-old *^1^ 0.50 Not living with a partner *^1^ 1.12 Low or medium educational level *	Took > 12 months to get pregnant *^1^ 0.57 Infections during pregnancy *^1^ 0.22 Extreme fatigue *^1^ 0.35	Previous depression *^1^ 0.92 Negative life events *^1^ 1.49 Family with a history of depression *^1^ 0.59
Soto-Balbuena et al. (2018) Spain [15]	Financial problems *^1^ 0.20 (2nd T)		-Changes in social relationships *^1^ 0.15–0.25 (all trimesters) -Stressful life events *^1^ 0.17 (1st T)
Sharapova et al. (2018) Switzerland [38] Immigrant women Native women	Socioeconomic status of the couple (low) *^1^ 0.30	Primiparity *^1^ 0.33	Trait anxiety *^1^ 0.59 Lack of satisfaction with marital support *^1^ 0.29 Trait anxiety *^1^ 0.73
Osnes et al. (2019) Norway [50]			Insomnia *^1^ 0.42
González-Mesa et al. (2019) Spain [37] Spanish women Turkish women	Lower educational level (state) *^1^ 0.23 Religion (state and trait) *^1^ 0.31–0.26 Unemployment (state) *^1^ 0.10	Increased number of children (state) *^1^ 0.17 Used assisted reproduction techniques *^1^ 0.15 (trait)	Low partner support (state and trait) *^1^ 0.21–0.17 Lack of interest from the couple in the pregnancy (state and trait) *^1^ 0.20–0.72
Cena et al. (2020) Italy [44]	Lower educational level * (<0.01) Unemployment* (<0.01) Economic difficulties* (<0.01)	Unplanned pregnancy * (<0.01) No use of assisted reproduction techniques * (<0.05) Abortion history * (<0.05) Multiparity * (<0.05)	
Insan et al. (2020) United Kingdom [41]			
British sample	No risk factors were found for the British sample.		
Asian sample	Older age *^1^ 0.12 Lower educational level *^1^ 0.65		
Savory et al. (2021) United Kingdom [42]			Previous psychiatric disorders *^1^ 3.95 Low self-efficacy *^1^ 1.27 Low family support *^1^ 1.13
Koyucu et al. (2021) United Kingdom [48]	Unemployment *^1^ 0.16	-Pregnancy Risks*^1^ 2.09	Less social support *^1^ 0.92
Filippetti et al. (2022) United Kingdom [40]			COVID-19 pandemic * [0.40, 0.69]
Răchită et al. (2023) Romania [47]	Live in an urban area *^1^ 1.84		

*: Predictor variables; ^1^: Odds ratio of predictor variables; (*p* value); ^r^: Correlation; []: Credible interval (Bayesian statistic); (state): Association with state anxiety; (trait): Association with trait anxiety; (state and trait): Association with state and trait anxiety; T: trimester.

### 3.3. Risk Factors for Prenatal Anxiety

The risk factors associated with prenatal anxiety will be described and organized into three categories: sociodemographic factors, obstetric and/or pregnancy-related factors, and psychological factors.

#### 3.3.1. Sociodemographic Risk Factors

Different sociodemographic factors have been evaluated in scientific literature (age, level of education, marital status, socioeconomic status, employment situation, social context, and residence in an urban or rural area. The most studied factor was the level of education (*n* = 10), followed by maternal age and socioeconomic status (*n* = 9), marital status (*n* = 8), employment situation and social context (*n* = 7), and place of residence (*n* = 2).

The age of the women, despite being one of the variables considered in most studies, was not always analyzed in relation to the presence of anxiety during pregnancy. The results obtained from the nine studies that examined this relationship are inconsistent. Specifically, only two studies [39,41] found a relationship between age and prenatal anxiety, and moreover, the relationships they found were opposite. Van de Loo et al. [39] found that being under 30 years old predicted the presence of anxiety during pregnancy. In contrast, Insan et al. [41] found a relationship with being older. The remaining seven studies [15,37,44,45,47,48,49] did not find any association between maternal age and prenatal anxiety.

Regarding the educational level, 10 out of the 13 studies reviewed have analyzed its relationship with prenatal anxiety. Among the five studies that have found a relationship, it has always been associated with a lower educational level [37,39,41,44,49].

Another factor that has been studied is socioeconomic status. In this case, out of the nine studies that analyzed this variable, four found a relationship in the same direction, meaning that being in an unfavorable socioeconomic situation is associated with the presence of prenatal anxiety [15,38,44,49]. Specifically, Sharapova et al. [38] found that it was the lower socioeconomic status of the partner that influenced the likelihood of experiencing anxiety during pregnancy, acting as a stressful risk factor and a predictor of prenatal anxiety in immigrant women.

A factor related to socioeconomic status is employment status. Of the seven studies that have analyzed the association between employment status and prenatal anxiety, three [37,44,48] have associated not having a job to women with prenatal anxiety. Specifically, Koyucu et al. [48] pointed out that pregnant women who had lost their jobs during the COVID-19 pandemic were three times more likely to develop anxiety during pregnancy. The other four studies found no relationship between the two variables.

Another factor that has been analyzed is marital status. In this case, only the study by van de Loo et al. [39] found a relationship between not cohabiting with a partner and a higher risk of experiencing prenatal anxiety. The remaining seven studies [15,37,38,42,44,45,47] found no association between marital status and prenatal anxiety. However, the findings also highlight the need to consider the quality of the relationship. In fact, Martini et al. [45] and Sharapova et al. [38] found that an unsatisfactory couple relationship was a predictor of prenatal anxiety.

The context of belonging of the pregnant woman, as well as the social environment in which she lives and socializes, has also been found to be a risk factor for experiencing anxiety during pregnancy. Out of the 13 studies included in this review, 7 analyzed the influence of context on prenatal anxiety levels. Specifically, four [15,37,39,42] examined the impact of belonging to an ethnic minority in the country of residence and/or being a foreigner, reporting that being foreign increased the likelihood of experiencing prenatal anxiety. Two studies [37,48] analyzed the influence of living with a larger number of people in the household, finding that a higher number of people in the household was associated with prenatal anxiety. Another two [41,47] investigated the effect of residing in urban or rural areas and/or socially deprived areas, and one [37] studied the effect of religion. Specifically, in Romania, it was found [47] that living in urban areas was associated with higher levels of prenatal anxiety compared to living in rural areas. In Turkey, it was found [37] that the Islamic religion, compared to other religions, was a predictor of prenatal anxiety.

Considering the previously discussed findings, the sociodemographic factors most consistently associated with prenatal anxiety are having a lower educational level and an unfavorable socioeconomic situation.

#### 3.3.2. Obstetric and/or Pregnancy-Related Risk Factors

In the studies included in this review, the influence of different obstetric and/or pregnancy-related risk factors (parity, history of miscarriages, pregnancy planning, time to conceive, medical problems during pregnancy, and undergoing fertility treatments) was evaluated. Parity has been the most studied pregnancy-related risk factor in relation to prenatal anxiety (*n* = 8), followed by complications and/or risks during pregnancy (*n* = 6), pregnancy planning (*n* = 5), previous miscarriages (*n* = 3), and fertility treatments (*n* = 2).

Regarding parity, five studies found no association [39,45,47,48,49] between the number of children and prenatal anxiety, while three studies did find an association [37,38,44]. Among these, the results were contradictory, as Cena et al. [44] and González- Mesa et al. [37] found that being multiparous was associated with prenatal anxiety, whereas Sharapova et al. [38] found that primiparity was associated with the presence of anxiety.

Six studies [15,37,39,44,47,48] analyze the relationship between prenatal anxiety and the previous and actual obstetric history of the women. Regarding the history of loss, only one study [44] found that it was a predictor for prenatal anxiety, whereas others [15,37] did not find this relation. The studies that analyzed the complications and/or risks in pregnancy [39,48] found an association between those and prenatal anxiety. Moreover, van de Loo et al. [39] found that taking more than 12 months to conceive was a predictor for prenatal anxiety.

Five studies evaluated the situation of an unplanned or unwanted pregnancy as a risk factor for antenatal anxiety [37,39,44,45,47]. Only one study [44] found that not planning the pregnancy was a predictor of prenatal anxiety.

Regarding fertility treatments, the data are contradictory, as the two studies [37,44] that investigated the relationship between undergoing fertility treatment and experiencing prenatal anxiety found opposing results. González-Mesa et al. [37] found that undergoing assisted reproductive methods was associated with higher levels of anxiety. In contrast, in the study by Cena et al. [44], using assisted reproduction techniques was not a risk factor.

Based on the mentioned findings, the presence of complications and/or risks during pregnancy is the variable most strongly associated with prenatal anxiety.

#### 3.3.3. Psychological Risk Factors

The psychological factors that have been studied regarding the risk of experiencing prenatal anxiety are primarily the presence of a history of mental health problems, social support, stressful life events, and personality characteristics. Among these, social support has been the most studied variable (*n* = 6). Additionally, four studies have provided results on the history of mental health problems, stress, and personality characteristics.

Four studies [39,42,45,50] analyzed the relationship between a history of mental health issues and prenatal anxiety. Specifically, a personal history of anxiety and/or depression [39,45] or psychopathological disorders in general [42,50] was associated with a higher risk of experiencing prenatal anxiety. Only one study examined the effect of the partner’s or family’s psychopathology [39], finding that a family history of depression increased the likelihood of experiencing anxiety symptoms at the beginning of pregnancy.

Regarding social support, most studies [15,37,42,45,48], except for one [38], agree that the absence or low perception of social support increases the risk of prenatal anxiety.

Concerning high levels of perceived stress during pregnancy and adverse life events, four studies [15,39,40,45] found that they played a significant role in the presence of anxiety. Specifically, van de Loo et al. [39] found that the average of symptoms of anxiety increased 1,5 and 1,8 at the beginning and at the end of the pregnancy when pregnant women faced negative life events. On the other hand, Soto-Balbuena et al. [15] concluded that stressful life events were the best predictors that explained the variance associated with anxiety during the three trimesters of pregnancy. Specifically, regarding social support, the results indicated an association between the frequency of seeing family members or friends and anxiety symptoms during the three trimesters. Meaning, the less one sees family or friends, the higher the likelihood of developing prenatal anxiety. On the other hand, Martini et al. [45] found an association with having a history of abuse. As for the COVID-19 pandemic, Filippetti et al. [40] found that women for whom COVID-19 had a greater psychological impact were more likely to experience symptoms of anxiety (95% HPDI = [0.40, 0.69]).

In relation to the psychological variables associated with prenatal anxiety, factors related to the woman’s personality are also found. Among the personality factors that increase the risk of experiencing anxiety during pregnancy, the anxiety trait, less effective coping strategies (e.g., crying and/or isolating vs. talking and/or seeking professional help), low self-esteem, and low self-efficacy stand out. Four studies analyzed these relationships. Specifically, Sharapova et al. [38] found that high punctuations in anxiety trait were predictors of prenatal anxiety in native women as well as in immigrants. Martini et al. [45] and Savory et al. [42] found an association between having low self-esteem or self-efficacy and prenatal anxiety. On the other hand, González-Mesa et al. [37] did not find an association between the type of coping strategies used and prenatal anxiety. Whereas they observed that women with low social support tended to use less effective coping strategies, such as crying in stressful situations.

## 4. Discussion

The objective of this review was to determine the risk factors associated with prenatal anxiety in women from Europe, as no studies on this topic had been conducted to date. It was found that numerous factors, including sociodemographic, obstetric, and/or pregnancy-related factors, as well as psychological factors, are associated with a higher risk of experiencing anxiety during pregnancy.

Regarding sociodemographic factors, having a lower educational level [15,44,49] or having an unfavorable socioeconomic situation [15,44,49] have been the variables that were consistently associated with prenatal anxiety in the European studies reviewed. Research conducted in other continents regarding educational level, such as the one carried out by Arifin et al. [51] in Malaysia or Bayrampour et al. [52] in Canada, found the same association. Likewise, a study conducted in Bangladesh [53] found that prenatal anxiety was inversely related to literacy. To interpret this finding, it should be considered that a low level of education is often associated with other disadvantages, such as low income [44]. Regarding socioeconomic status, our findings are also consistent with those from other continents, such as Canada [52] or China [10]. It has been suggested that during pregnancy, a low socioeconomic status is often accompanied by greater stress related to financial difficulties, which in turn is a risk factor for prenatal anxiety [54]. Similarly, Sharapova et al. [38] found that a lower socioeconomic status of the partner influenced the likelihood of experiencing anxiety during pregnancy, acting as a stressful risk factor. However, it should be noted that it was a predictor of prenatal anxiety specifically in immigrant women. A possible explanation for this finding is that, in the context of migration, partner support becomes even more important [55]. Additionally, in this study, immigrant women expressed fears of losing their jobs after the birth of their baby and, consequently, facing financial difficulties. This was particularly the case for women whose legal residence in the country was tied to their employment status, meaning that having a job was necessary for them to be able to stay in the country.

Regarding employment status, the studies that have found a relationship have found it in the same direction. Specifically, not having a paid job has been associated with prenatal anxiety in Spain [37], Italy [44], and the United Kingdom [49]. In Saudi Arabia [56], the same association was found. A possible explanation could be that not working implies having a smaller social support network and leads to a certain level of isolation [57]. However, in this case, cultural factors may play a role in this relationship. For example, in Pakistan, the opposite was found [58], meaning that working was a risk factor for prenatal anxiety. To interpret this finding, it is important to consider that in Pakistani families, working women are highly stigmatized, as the home is considered the proper place for women, just as being an obedient wife and a loving mother is expected.

The rest of the sociodemographic variables analyzed, such as age, marital status, or social context, have shown contradictory results and/or have been investigated in only a few studies that found an association with prenatal anxiety. Therefore, it is difficult to draw conclusions and compare them with findings from other countries. Regarding women’s age, the results in this review were inconsistent, as some studies found a significant relationship between younger age and experiencing anxiety during pregnancy [39] and others found the opposite [41]. This discrepancy coincides with the data found in other continents, such as, for example, Bayrampour et al. [52] in Canada, and Qiao et al. [59] in China, where they found an association between being younger and prenatal anxiety; it has been attributed to the fact that a young age hinders economic and emotional stability to face economic and/or family problems [60]. On the contrary, Dunkel-Schetter et al. [61] in Canada and Ali et al. [62] in Pakistan found the opposite association. As a possible explanation of this association about being older has been explained due to the possible difficulties conceiving, added to the possible anxiety due to pregnancy complications related to the mother’s age [63,64]. On the other hand, other European studies [15,44] and non-European [65,66] pointed out that age is not associated with anxiety in the prenatal phase. Therefore, more than the age itself, the explanation for this inconsistency can be related to cultural aspects, such as that in some societies being a young mother is the norm and what is expected, while in others, the opposite occurs.

Regarding obstetric and/or pregnancy-related factors, the presence of complications and/or risks in the current pregnancy is the variable that has received the most support in the studies reviewed [39,48,49]. This aligns with data found in Pakistan [62] and in Canada [61]. A possible explanation could be that these events could be very stressful and consequently increase the probability of developing prenatal anxiety [49]. Similarly, the presence of physical symptoms or pregnancy-related risks has also been associated with prenatal anxiety among European women [39,48]. For example, in the Netherlands, extreme fatigue was found to be a predictor of prenatal anxiety [39], and in the United Kingdom, researchers [48] found that women experiencing obstetric risks during pregnancy were twice as likely to suffer from prenatal anxiety. Outside of Europe, a higher likelihood of prenatal anxiety was also observed among Chinese women with anemia and/or pregnancy-induced hypertension syndrome [67], as well as among Canadian women [61] with certain medical risks (e.g., gestational diabetes, bleeding, or hospitalization during gestation).

Regarding the remaining obstetric and/or pregnancy-related variables, no definitive conclusions can be drawn, as the lack of pregnancy planning has only been associated with prenatal anxiety in one study. However, this finding aligns with those observed in other countries, such as Saudi Arabia [56], Qatar [68], Brazil [69], or Rwanda [66]. Regarding parity and the use of fertility treatments has only been associated with prenatal anxiety in three and two studies, respectively, and the results are contradictory. Regarding parity, contradictions were also found in other countries around the world. For example, in China [5] and in Canada [52] found that nulliparity was associated with prenatal anxiety. However, in a review conducted in Canada [70], they found the opposite, that is, being multiparous increased the risk of prenatal anxiety. Regarding fertility treatments, no studies outside of Europe identified a relationship between undergoing fertility treatments and prenatal anxiety.

With respect to psychological risk factors, the reviewed studies show a consensus that a history of mental health issues, high levels of stress and/or exposure to stressful life events, and low social support increase the risk of experiencing anxiety during the prenatal stage. Woman’s history of mental health problems, particularly having suffered from anxiety and/or depression, has been associated with a higher risk of anxiety both in European countries [39,44,45], as outside Europe, as shown in the studies conducted in Mexico [65], Canada [52] or Qatar [68].

Regarding social support, the findings in this review coincide with those found in non-European countries, indicating that the lack or low perception of social support [15,42], and specifically in the partner [37], increases the risk of prenatal anxiety. In Canada [61], Mexico [65], or India [71], the same association was found. In this sense, family contexts are important, even if they are very different among different cultures [37]. On the other hand, nuclear family environments are common in occidental countries, whereas extended family structures are more common in other places, such as Asia and/or Arab countries [72]. Some authors [51,54] found that women living in a nuclear family environment were at a higher risk of developing prenatal anxiety compared to those in a multigenerational household. This may be because women in nuclear families feel isolated and receive less social support than they would in a broader family setting. Conversely, living in an extended family has been identified as a protective factor against prenatal anxiety. However, González-Mesa et al. [37] found the opposite, as women living in larger families, with more children and more relatives in the same household, were at a higher risk of experiencing antenatal anxiety. Of these women, 40.0% reported having insufficient family support, despite the common belief in the strength of traditional family relationships.

Four studies analyzing personality traits have been found; however, the results are inconclusive, as each study examined a different variable (anxiety trait, less effective coping strategies, low self-esteem, and low self-efficacy). However, it is worth noting that studies conducted outside of Europe, as in China [10], reported that self-esteem was the best predictor of prenatal anxiety at all stages of pregnancy, indicating that women with low self-esteem might feel less prepared to face the challenges and stress of pregnancy. Regarding coping strategies, optimism stands out. In Canada, it was found [52] those pregnant women who were less optimistic had a four times higher risk of developing chronic anxiety symptoms. Optimism could reduce stress by influencing coping strategies when facing stressors.

When interpreting the obtained results, it is important to consider the culture of each country, as the European continent is made up of countries with very different cultures and socioeconomic levels. Cultural differences may explain part of the variability found in some of the risk factors, as it must be kept in mind that there are significant differences in areas such as the type of prenatal care women receive and the ease of access to it, the professionals overseeing the pregnancy (gynecologists or midwives), attitudes toward pregnancy and motherhood, and/or the role of women in decisions regarding their pregnancy.

Independently of cultural variability, based on the previous discussion, we can address the second objective of this review, which is to determine whether the risk factors associated with prenatal anxiety in European women are the same as those in women from other continents. The results suggest that they are, as the studies reviewed from non-European countries show that the variables associated with prenatal anxiety in those countries are similar to those found in Europe. Furthermore, they coincide on the factors where contradictory results have been found (e.g., parity, marital status, and age), or those that have been less studied (e.g., personality traits and fertility treatments). It can also be observed that psychological variables are the ones most consistently associated with anxiety across all countries worldwide. This is highly significant, as these are potentially modifiable factors through appropriate intervention. Therefore, there is a need to develop preventive intervention protocols at the psychological level, e.g., [73], to reduce the likelihood of developing anxiety during this stage and the associated consequences.

Lastly, to answer the question whether prenatal anxiety-related factors are associated with the ones of prenatal depression, we took as reference a review carried out in recent years [30] where risk factors associated with prenatal depression worldwide between 2010 and 2020 are analyzed. Regarding sociodemographic factors, it can be observed that the findings of these authors align with those found in our review, as a lower educational level and a worse socioeconomic situation have been associated with prenatal depression. Regarding obstetric variables, Míguez and Vázquez [30] found that having a non-planned pregnancy was the factor most associated with depression. This finding is consistent with the one found in Italy by Cena et al. [44] regarding prenatal anxiety. However, obstetric and/or pregnancy-related complications were the factors that were most strongly associated with prenatal anxiety in this study. A possible explanation could be that only three studies evaluated the relationship between prenatal anxiety and pregnancy planning, compared to seven that considered pregnancy complications and/or risks. Finally, the findings of this study regarding psychological factors align with those found for prenatal depression, as a history of mental health issues, stressful life events, and low social support during pregnancy were predictors of prenatal depression. Considering this, it is evident that the vast majority of risk factors associated with prenatal anxiety are also linked to prenatal depression (Table 3). This finding is highly relevant, as allocating resources to identifying these risk factors can have an impact on both conditions. Moreover, a single intervention could help prevent the onset of both anxiety and depression during pregnancy. A promising type of intervention would be cognitive behavioral therapy, since this approach includes components that would act on various modifiable risk factors. For example, training in communication skills and problem solving could help improve social support or deal more appropriately with problematic situations; emotion management and behavioral activation would allow us to have strategies to adequately cope with stress, anxiety or depressed mood, as well as cognitive restructuring to generate alternatives to irrational thoughts that increase emotional discomfort during this stage.

Some limitations of this study should be considered when interpreting the findings. First, the review is limited to studies published in English, so the generalization of the findings is limited. This criterion has been included because although there is a great diversity of languages on the European continent, most of the scientific literature of the countries is published in English. Another aspect to take into account when generalizing the data is that in some of the samples, there was a minority representation of immigrant women and/or women of different ethnicities than European women (e.g., 37, 41, and 42). Second, the heterogeneity in the included studies, such as the type of design (longitudinal, cross-sectional, or cohort), sample size, or the type of analysis (bivariate vs. multivariate), has hindered the ability to perform a meta-analysis of the findings, which could have provided additional information on the differential impact of each risk factor. On the other hand, the heterogeneity of the assessment instruments, added to the fact that most studies assessed anxiety using self-report measures (only one study used a clinical interview) and with different cut-off points, is an important factor to consider when identifying the risk factors associated with prenatal anxiety and consequently poses some sort of limitation when generalizing the results. In this regard, it should be remembered that self-report is a screening measure and may result in false positives; therefore, it may identify risk factors that would not be found when using a clinical interview. Related to this, some factors were associated with generalized anxiety disorder (e.g., unsatisfactory relationship) and not with anxious symptomatology, or had stronger associations with generalized anxiety disorder (e.g., previous history of anxiety/depression) and not with general anxiety as assessed by self-report. Likewise, the existing controversy regarding the definition of anxiety is also an important aspect to take into account, as the findings may vary depending on what each assessment instrument understands as anxiety. This could lead to the identification of different risk factors depending on the assessment instrument used. Additionally, this review excluded studies prior to 2015 and those based on high-risk populations (e.g., studies conducted on women with pre-existing conditions, living in poverty situations, experiencing high-risk or twin pregnancies, or being victims of gender-based violence). Therefore, the generalization of the findings to these populations may be limited.

## 5. Conclusions

This review shows that there are multiple factors that contribute to women experiencing prenatal anxiety; furthermore, many of these appear to be similar to those associated with prenatal depression. Most of these can be identified early in pregnancy during prenatal visits (e.g., socioeconomic or educational level; pregnancy complications). Additionally, some factors, such as psychological ones, are potentially modifiable through appropriate interventions. This highlights the importance of conducting proper screenings for anxiety during pregnancy to prevent its onset or to treat it adequately if necessary. Furthermore, the fact that risk factors are common for both anxiety and prenatal depression provides promising findings, suggesting that a single intervention could reduce the likelihood of the occurrence of both conditions and their possible consequences.

## Figures and Tables

**Table 3:** Risk factors for prenatal anxiety and depression.

	Anxiety	Depression
**Sociodemographic factors**		
Lower educational level	V	V
Socioeconomic status	V	V
**Obstetric factors**		
Unplanned pregnancy		V
Complications and/or risks during pregnancy	V	
**Psychological factors**		
History of psychological disorders	V	V
Prenatal depression		V
Presence anxiety		V
Exposure to adverse life events	V	V
Low social support	V	V

## Data Availability

Not applicable.

## Supplementary Materials

The following supporting information can be downloaded at: https://www.mdpi.com/article/10.3390/jcm14093248/s1, Table S1: Details of Search Strategy for Each Database.
