# Supplementary material for: Risk Factors for Prenatal Anxiety in European Women: A Review
Source: J Clin Med. 2025 May 7;14(9):3248. doi: 10.3390/jcm14093248 (PMC12072564; doi:10.3390/jcm14093248)
Supplement: Supplementary file 1 [file jcm-14-03248-s001.zip › jcm-3590136-supplementary.pdf]

Table S1. Details of Search Strategy for Each Database

**Database**	**Search, terms, connector, truncation uses**
PsycINFO	tiab(antenatal anxiety) OR tiab(anxiety during pregnancy) AND tiab(risk factors) AND tiab(variables associated)<br>Limits: date and english language
MEDLINE	XB antenatal anxiety OR XB anxiety during pregnancy AND XB (risk factors OR variables associated)<br>Limits: date and english language
SCOPUS	TITLE-ABS-KEY (("antenatal anxiety" OR "anxiety during pregnancy" AND "risk factors" OR "variables associated")) AND PUBYEAR > 2014 AND PUBYEAR < 2025 AND (LIMIT-TO (LANGUAGE , "English"))
